# The EAT-Lancet diet in relation to nutrient intake among older adults: insights from the Gothenburg H70 birth cohort study

**DOI:** 10.1186/s12937-025-01193-7

**Published:** 2025-08-08

**Authors:** Anna Stubbendorff, Silke Kern, Lina Rydén, Ingmar Skoog, Jessica Samuelsson

**Affiliations:** 1 Department of Clinical Sciences Malmö, Lund University, Jan Waldenstöms gata 35, Malmö, 21428 Sweden; 2 Neuropsychiatric Epidemiology Unit, Department of Psychiatry and Neurochemistry, Institute of Neuroscience and Physiology, Sahlgrenska Academy, Centre for Ageing and Health (AGECAP) at the University of Gothenburg, Wallinsgatan 6, Mölndal, 43139 Sweden; 3 Region Västra Götaland, Department of Neuropsychiatry, Sahlgrenska University Hospital, Gothenburg, Sweden

**Keywords:** Sustainable diet, Plant-based diets, Nutrient deficiency, Aging

## Abstract

**Background:**

The EAT-Lancet Commission has proposed a global reference diet aimed at promoting both human health and environmental sustainability. While adherence to this dietary pattern has been associated with reduced risks of chronic disease and lower environmental impact, concerns remain about its ability to meet nutritional requirements - particularly among older adults. The aim was to explore the association between adherence to the EAT-Lancet diet and nutrient intake and adequacy among 70-year-old adults in Gothenburg, Sweden.

**Methods:**

This cross-sectional study included 861 participants from the Swedish population-based Gothenburg H70 Birth Cohort Study (mean age 70.5 years, 55% women). Dietary intake was assessed using a validated diet history interview, and adherence to the EAT-Lancet diet was scored based on 14 food components. Nutrient intake was evaluated against age- and sex-specific recommended intake (RI) levels. Cardiometabolic risk markers and biomarkers of nutritional status, including homocysteine and haemoglobin, were measured. Linear and logistic regression models were used to examine trends across sex-specific tertiles of diet adherence, with sensitivity analyses adjusting for energy intake and comparing adequacy based on average requirement (AR) thresholds.

**Results:**

Higher adherence to the EAT-Lancet diet was linked to higher intake of fibre and polyunsaturated fats, and lower intake of saturated fat and alcohol. Mean protein intake per kilogram body weight/day was similar across adherence tertiles. Intake of beta-carotene, folate, vitamin C, magnesium, potassium, and iron was higher with greater adherence, while retinol equivalents, vitamin B12, niacin equivalents was lower– patterns that remained consistent after energy adjustment. Despite lower B12 intake, homocysteine levels were lowest in the group with highest adherence, and anaemia prevalence did not differ. Micronutrient adequacy improved with higher adherence for vitamin E, folate, vitamin C, magnesium, potassium, and iron. Similar results were observed using average requirement (AR) thresholds in sensitivity analyses.

**Conclusions:**

Adherence to the EAT-Lancet diet was associated with a more favourable nutrient profile in this cohort of older adults, without evidence of widespread micronutrient inadequacy. These findings suggest that environmentally sustainable diets can support adequate nutrition when well-balanced, even in nutritionally vulnerable populations such as older adults.

**Supplementary Information:**

The online version contains supplementary material available at 10.1186/s12937-025-01193-7.

## Introduction

Current dietary habits pose risks to both human health and environmental sustainability [1]. Food production is a major driver of climate change, contributing to approximately one-third of global anthropogenic greenhouse gas emissions, 40% of land use, 70% of freshwater consumption, and major biodiversity loss [1–4]. Diets high in animal-sourced foods have up to four times the environmental impact of plant-based diets in terms of emissions and land use, nearly triple the effect on biodiversity, and twice the water consumption [5]. A transition toward more plant-based, sustainable diets is therefore essential for mitigating environmental damage and achieving the United Nations’ Sustainable Development Goals [6, 7].

In response to the demanding health and environmental challenges, the EAT-Lancet Commission introduced the “planetary health diet” in 2019, a reference diet aimed at promoting both human health and environmental sustainability [1]. This diet emphasizes plant-based foods such as whole grains, vegetables, fruits, legumes, nuts, and unsaturated fats, while limiting added sugar, dairy, red meat, and other animal-based foods. It was designed to reduce the burden of non-communicable diseases and malnutrition while simultaneously minimizing the environmental impact of food systems.

Adherence to the EAT-Lancet diet has been linked to a lower environmental footprint [8, 9] and improved health outcomes, including reduced risks of mortality, cardiovascular disease, type 2 diabetes, and cancer [8, 10–12]. Further evidence suggests that greater adherence may lower the risk of stroke [13], coronary events [12], brain health and cognitive decline in old age [14–16]. These findings highlight the potential of the EAT-Lancet diet as a sustainable dietary framework to improve both population health and planetary well-being.

Given the increased focus on incorporating environmental sustainability into general dietary guidelines, it is important to understand how planetary health diets, such as the EAT-Lancet diet, impact micronutrient intake and risk of micronutrient deficiencies. While the diet has been linked to improved health outcomes, concerns remain about the diet’s ability to provide sufficient micronutrients, an important factor to consider as over two billion people worldwide affected by micronutrient deficiencies [17–20]. However, to date, only a few studies have assessed nutrient adequacy of self-reported diets in relation to the EAT-Lancet diet [21–26], and only one study has assessed the impact in older adults in particular [27]. Exploring this among older adults is especially important, as the risk of malnutrition increases with age [28]. The aim of this study was to assess adherence to the EAT-Lancet diet in relation to nutrient intake among 70-year-olds in Gothenburg, Sweden.

## Methods

### Study design and participants

This study used cross-sectional data from the Swedish population-based Gothenburg H70 Birth Cohort Study (H70 study), conducted between 2014 and 2016 [29]. The selection process targeted all individuals born in 1944 who were residents of Gothenburg and had birth dates ending in 0, 2, 5, or 8. Of those invited, 1203 individuals participated, resulting in a 72% response rate. The systematic selection strategy aimed to ensure a diverse and representative sample in terms of equity and inclusion. The study included a comprehensive one-day examination at the Neuropsychiatric Clinic at Sahlgrenska University Hospital and additional examinations. The study comprised sampling of blood and cerebrospinal fluid, psychiatric, cognitive, and physical health examinations, examinations of genetics and family history, use of medications, social factors, functional ability, physical activity, body composition, lung function, audiological and ophthalmological examinations, diet, and brain imaging [29]. All participants were invited to take part in the dietary assessment. Exclusion criteria was inability to remember and communicate dietary intake (e.g., dementia). Of these, 861 individuals completed the dietary assessment [30]. Reasons for non-participation varied and have been previously documented [29, 30]. A participant flowchart is provided in supplemental Fig. 1.

### Dietary examination and nutrient calculation

The dietary assessment in this study was conducted using a diet history method, which in this study consists of a semi-structured, face-to-face interview carried out by registered dietitians. This approach aimed to estimate habitual food intake over the previous three months and has been described in detail elsewhere [29, 30]. The interview protocol included a meal-pattern assessment, where participants provided details on the types of foods consumed, their usual frequency, and portion sizes. To assist in estimating portion sizes, visual aids from the Swedish Food Agency (SFA) were used. Reported food consumption was recorded in grams per day, week, or month using the Dietist Net Pro software (Kostdata AB, Bromma), which incorporates the SFA’s 2015 nutrient database [30].

Mean daily food intake was estimated based on results from the diet history interview, and nutrient intake was based on dietary intake without inclusion of dietary supplements. Protein, fat, and carbohydrate intakes were calculated as percentages of non-alcoholic energy intake (E%). To calculate protein intake per kilogram (kg) of body weight (bw), an adjusted weight was used for individuals with a BMI above 25 to approximate a healthier reference level. Specifically, the adjusted weight was defined as the weight corresponding to a BMI of 25, plus 25% of the excess weight (i.e., the difference between the individual’s actual weight and the weight corresponding to a BMI of 25). This approach aimed to account for the potential influence of excess body weight while still reflecting individual variation. Micronutrient adequacy from diet was evaluated against age- and sex-specific reference values for recommended intake (RI) for individuals aged > 70 years, based on the Nordic Nutrition Recommendations 2023 [31]. We additionally used the average requirement (AR) as a cut-off to estimate the proportion of participants at risk of inadequate intake at a lower threshold than the recommended intake. To reflect differences in bioavailability and physiological effects, conversions were applied for several nutrients. One retinol equivalent (RE) was defined as 1 µg of preformed vitamin A (retinol) from diet or supplements, 2 µg of supplemental β-carotene, 6 µg of dietary β-carotene, or 12 µg of other provitamin A carotenoids such as α-carotene and β-cryptoxanthin. Niacin equivalents (NE) were used to account for both preformed niacin (vitamin B3) and niacin synthesized from tryptophan, with 60 mg of tryptophan considered equivalent to 1 mg NE.

### Blood sampling

As a complement to the descriptive baseline characteristics, biomarkers including homocysteine, haemoglobin, glucose, and cardiometabolic risk factors (total cholesterol, HDL-cholesterol, LDL-cholesterol, and triglycerides) were analysed. Out of the 861 participants with dietary data, blood sampling was performed on 857 participants (after overnight fasting for 834 participants). A maximum of 120 ml blood was drawn, of which 15 ml was used for blood tests. A maximum of 105 ml was aliquoted into serum (after 20–30 min of coagulation, 5 ml SST tubes were centrifuged with 2000 RCF (Relative Centrifugal Force) for 10 min and then pipetted into 1.5 ml micro tubes), plasma (10 ml EDTA tubes were centrifuged with 2000 RCF (Relative Centrifugal Force) in 20 °C for 15 min and then pipetted into 1.5 ml micro tubes) and whole blood (10 ml EDTA tubes pipetted into 1.5 ml micro tubes) and were frozen at − 80 °C within approximately one hour to be saved in a biobank for future analyses according to the Swedish Biobank Law (2002:297) (biobank registration date: 2005-03-17; Registration Number: 532 at the National Board of Health and Welfare). All study participants gave written consent regarding biobanking. Blood tests included, haemoglobin (Hb, *n* = 850), glucose (*n* = 851), lipids (total-, HDL- and LDL-cholesterol, triglycerides, *n* = 855) and indirect measurement of vitamin B12 and folic acid status (homocysteine, *n* = 836) [29]. Reference values were used from Västra Götaland Regional Council [32].

### The EAT-Lancet diet score

The EAT-Lancet diet score applied in this study was adapted from the scoring system developed by Stubbendorff et al. in 2022 [10], which is based on the dietary recommendations proposed by the EAT-Lancet Commission in 2019 [1]. This score has performed well in a systematic comparison to other EAT-Lancet scores in cohorts from Denmark, Sweden and Mexico [9]. Reported dietary intake was categorized into specific food components, expressed in grams per day based on uncooked weight (Supplementary Table 1). The score is built on the principle of classifying food into two categories: “emphasized foods” and “limited foods”. The emphasized category included vegetables, fruits, legumes, whole grains, nuts, fish, and unsaturated fats, while the limited category comprised beef and lamb, pork, poultry, eggs, dairy, potatoes, and added sugar. In this cohort we used unsaturated fat as a nutrient instead of the food sources of unsaturated fat. The index consists of 14 food components, with each assigned a score ranging from 0 to 3 points. A score of 0 reflects low adherence, whereas a score of 3 indicates high adherence to the EAT-Lancet dietary recommendations (Supplementary Table 1). The total index score ranges from 0 (no adherence) to 42 (optimal adherence, 14 × 3 points).

### Characteristics of potential confounders

Data on participant characteristics and potential confounders were collected through semi-structured interviews and health examinations, as previously described [29]. Age was not added as a confounder as all participants were examined at age 70–71 years. Sex assigned at birth was identified by the Swedish personal identity number. A Swedish personal identity number is a unique identification number allocated to a person when they are born, or when they move to Sweden. Educational level was divided into three categories based on Swedish education classifications: compulsory primary education (pre-primary education, primary and lower secondary education less than 9 years), secondary education (primary and lower secondary education 9 years, upper secondary education, and post-secondary education less than 2 years), and higher education (post-secondary education 2 years or longer, and postgraduate education). Smoking was defined as never smoked, previous smoker (before 50 years, between 50 and 60 years, or between 60 and 70 years), and current smoking. Physical activity was divided into four categories: physically inactive, some light physical activity, regular physical activity and training (1–2 h/week), and regular more intense physical training (> 3 h per week). Diabetes was measured as diabetes yes/no. Hypertension was measured as ≥ 140/90 mmHg (yes/no).

### Statistical analysis

Tertiles of adherence to the EAT-Lancet diet was created by sex assigned at birth. Baseline characteristics of all participants were presented according to tertiles of adherence. Continuous variables were reported as means with standard deviations (SDs), while categorical variables were expressed as frequencies and percentages. Pearsons’s correlation coefficients between food groups in the EAT-Lancet diet macronutrients and micronutrients were illustrated by a correlation matrix (heatmap). Intake of macronutrients and micronutrients were reported across EAT-Lancet diet adherence tertiles, based on linear regression analyses with predicted margins, and p-values for trend. Nutrient adequacy based on RI was evaluated using logistic regression with predicted margins, and p-values for trend were reported. Comparisons of biomarker status by tertiles of adherence to the EAT-Lancet diet was performed with linear regression models.

All statistical analyses were conducted using STATA software (version 18.0, StataCorp, USA). A two-sided p-value of < 0.05 was considered statistically significant.

### Sensitivity analyses

As a sensitivity analysis, we adjusted micronutrient intakes for total energy intake. In addition, we evaluated nutrient adequacy based on Average Requirement (AR) as sensitivity analyses to further explore the risk of nutrient deficiencies of adhering to the EAT-Lancet diet. For biomarker analyses, we performed stepwise adjustments by sequentially including the covariates education, physical activity, smoking, and BMI. Since BMI may act as a mediator rather than purely a confounder, results are described both with and without BMI adjustment.

## Results

### Characteristics of participants and adherence to the EAT-Lancet diet

A total of 861 participants, with a mean age of 70.5 (SD 0.26) years, were included in the study (Supplemental Fig. 1), of which 55.1.5% were women. Participants in the highest tertile of adherence to the EAT-Lancet diet were more likely to have a lower BMI (25.0 kg/m²) compared to those in the lowest tertile (26.6 kg/m²) (Table 1). Participants in the highest tertile were more physically active and less likely to be inactive compared to those in the lowest tertile. Higher education was more common in the highest tertile, while current smoking was less prevalent.

**Table 1:** Baseline characteristics according to the sex-specific tertiles of the EAT-Lancet diet score in the H70 study

	Baseline characteristics across tertiles of EAT-Lancet diet score^1^				All
	T1: 10–21	T2: 21–24	T3: 24–38	*p*-value	
n	322 (37.4%)	284 (33.0%)	255 (29.6%)		
BMI	26.6 (5.0)	25.9 (4.3)	25.0 (4.0)	< 0.001	25.9 (4.5)
Physical activity					
Inactive	17 (5.3%)	3 (1.1%)	4 (1.6%)	< 0.001	24 (2.8%)
Light physical activity	54 (17.0%)	19 (6.8%)	22 (8.8%)		95 (11.2%)
Regular physical activity and training	158 (49.7%)	136 (48.7%)	111 (44.2%)		405 (47.8%)
Regular and more intense physical training	89 (28.0%)	121 (43.4%)	114 (45.4%)		324 (38.2%)
Education					
< 9 years	33 (10.3%)	26 (9.2%)	10 (3.9%)	0.001	69 (8.0%)
Secondary	152 (47.4%)	132 (46.5%)	99 (38.8%)		383 (44.5%)
Higher education	136 (42.4%)	126 (44.4%)	146 (57.3%)		408 (47.4%)
Smoking status					
Never smoked	110 (34.4%)	125 (44.2%)	105 (41.2%)	< 0.001	340 (39.6%)
Stopped smoking before 50 y	87 (27.2%)	105 (37.1%)	90 (35.3%)		282 (32.9%)
Stopped smoking between 50–60 y	34 (10.6%)	34 (12.0%)	35 (13.7%)		103 (12.0%)
Stopped smoking between 60–70 y	41 (12.8%)	13 (4.6%)	12 (4.7%)		66 (7.7%)
Current smoker	48 (15.0%)	6 (2.1%)	13 (5.1%)		67 (7.8%)
Diabetes	24 (7.5%)	35 (12.3%)	20 (7.8%)	0.083	79 (9.2%)
Hypertension	232 (72.3%)	200 (70.4%)	169 (66.3%)	0.288	601 (69.9%)

^1^Values are means (SD) for continuous variables and n (%) for categorical variables

### **Adherence to the EAT-Lancet diet and food intake**

Among women, the mean EAT-Lancet diet score was 23.1 (± 3.8), compared to 21.5 (± 3.6) for men. Higher adherence to the EAT-Lancet diet was associated with greater intake of whole grains, vegetables, fruits, legumes, nuts, and seafood (Table 2). Conversely, the consumption of potatoes, beef, lamb, pork, poultry, eggs, dairy products, and added sugars decreased across tertiles of adherence.

**Table 2:** Food intake per day across sex-specific tertiles of the EAT-Lancet diet score in the H70-study

	Food intake across tertiles of EAT-Lancet diet score^1^				All
	T1: 10–21	T2: 21–24	T3: 24–38	p-trend^2^	
Whole grain	96.8 (59.8)	117.6 (64.1)	123.6 (60.3)	< 0.001	111.6 (62.5)
Potatoes	126.6 (87.8)	89.6 (68.4)	61.9 (60.1)	< 0.001	95.2 (78.7)
Vegetables	131.4 (80.1)	192.8 (97.6)	248.2 (119.3)	< 0.001	186.3 (109.6)
Fruit and berries	168.4 (140.9)	251.9 (149.1)	303.0 (163.1)	< 0.001	235.8 (160.4)
Milk equivalent	685.8 (379.3)	640.6 (328.3)	612.4 (292.7)	< 0.001	649.2 (339.7)
Beef and lamb	34.7 (19.9)	29.7 (17.4)	19.3 (18.9)	< 0.001	28.5 (19.8)
Pork	50.5 (28.6)	42.2 (24.5)	25.3 (28.3)	< 0.001	40.3 (29.1)
Poultry	21.6 (24.9)	20.6 (17.0)	18.9 (16.5)	0.103	20.5 (20.2)
Egg	32.4 (24.9)	27.2 (21.6)	21.8 (19.5)	< 0.001	27.6 (22.7)
Seafood	60.2 (38.0)	70.4 (41.9)	72.9 (40.2)	< 0.001	67.3 (40.3)
Legumes	7.2 (7.3)	9.7 (11.2)	17.7 (20.8)	< 0.001	11.1 (14.4)
Nuts and seeds	5.5 (8.4)	10.6 (15.1)	19.2 (22.3)	< 0.001	11.2 (16.7)
Added sugar	50.0 (30.3)	39.2 (19.8)	32.8 (17.5)	< 0.001	41.4 (24.8)
Unsaturated fat	44.0 (13.9)	45.8 (14.4)	47.8 (14.2)	< 0.001	45.7 (14.2)

^1^Values are mean intake per g/day and (SD)

^2^P-value for linear trend

### Food consumption and nutrient intake

**Fig. 1:**
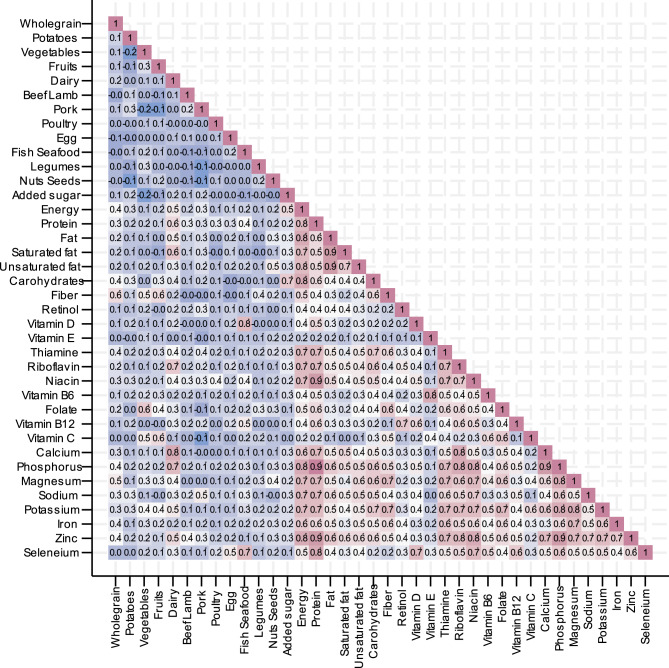
Correlation matrix of food groups, macronutrients, and micronutrients in the H70 study. The heatmap displays Pearson correlation coefficients between food group intake and nutrient intake

A correlation matrix of all included foods and nutrients can be found in Fig. 1. Beef, lamb, pork and poultry correlated positively with protein, while milk and dairy products were strongly linked to calcium and riboflavin. Fish and seafood showed strong correlations with vitamin D, polyunsaturated fatty acids, and selenium. Added sugar intake was negatively correlated with fibre, beta-carotene, folate, magnesium, and vitamin C. Saturated fat was associated with dairy and red meat, while polyunsaturated fats were correlated with nuts, seeds, and fish. Vitamin B12 was positively correlated with intake of animal-based foods such as meat, fish, and eggs. Folate and fibre were more closely linked to plant-based foods, including whole grains, legumes, and vegetables. Iron was positively associated with both animal sources, such as red meat, and plant-based sources, including legumes and whole grains.

### Nutrient intake

A higher adherence to the EAT-Lancet diet measured from T1 to T3 was associated with higher intake of dietary fibre and polyunsaturated fatty acids (Table 3). Conversely, higher adherence was associated with a lower intake of protein, alcohol, and saturated fatty acids. However, there was no difference in protein intake when measured per kg bw/day across tertiles, and mean intake was in line with recommended intake for older adults of 1.2 g/kg/bw/day. No significant trends were observed for total energy intake or other macronutrients.

**Table 3:** Nutrient intake per day across sex specific tertiles of EAT-Lancet diet score H70-study

	Nutrient intake across tertiles of EAT-Lancet diet score^1^			
	T1: 10–21	T2: 21–24	T3: 24–38	p-trend^2^
Energy (kcal/day)	2180 (571)	2156 (486)	2128 (437)	0.221
Fat (g/day)	88.9 (28.4)	88.6 (27.2)	88.1 (24.7)	0.727
E% fat^2^	38.4 (6.5)	38.3 (7.0)	38.5 (6.2)	0.773
Saturated fatty acids (g/day)	37.4 (14.0)	35.5 (12.9)	33.2 (11.9)	< 0.001
E% SFA^3^	16.1 (3.7)	15.3 (3.8)	14.4 (3.6)	< 0.001
Monounsaturated fatty acids (g/day)	31.7 (10.2)	31.8 (10.3)	32.1 (9.6)	0.642
E% MUFA^3^	13.7 (2.5)	13.7 (2.8)	14.1 (2.8)	0.129
Polyunsaturated fatty acids (g/day)	12.3 (4.7)	14.0 (5.6)	15.7 (6.4)	< 0.001
E% PUFA^3^	5.3 (1.6)	6.1 (2.1)	6.9 (2.5)	< 0.001
Carbohydrates (g/day)	220 (73.2)	216 (61.3)	214 (54.9)	0.245
E% carbohydrates^3^	42.0 (6.8)	41.6 (6.7)	41.7 (6.0)	0.589
Fiber (g/day)	21.9 (7.2)	26.1 (7.8)	30.3 (8.4)	< 0.001
Protein (g/day)	89.3 (23.3)	88.9 (21.0)	85.4 (20.4)	0.038
Protein (g/kg body weight)^4^	1.26 (0.36)	1.24 (0.33)	1.24 (0.30)	0.433
E% protein^2^	17.4 (3.0)	17.4 (3.1)	16.7 (2.6)	0.006
Alcohol (g/day)	13.8 (17.0)	12.1 (13.3)	10.9 (10.5)	0.013
E% Alcohol^5^	4.4 (5.1)	3.9 (4.2)	3.6 (3.4)	0.033
Retinol Equivalent	1204 (697)	1125 (618)	1057 (650)	0.008
Beta-Carotene (µg/day)	2779 (1964)	3441 (2473)	4415 (4020)	< 0.001
Vitamin D (µg/day)	8.6 (3.8)	9.1 (4.3)	8.9 (4.2)	0.335
Vitamin E (mg/day)	15.4 (15.5)	16.4 (15.0)	15.6 (11.1)	0.787
Thiamine (mg/day)	1.4 (0.4)	1.4 (0.4)	1.4 (0.4)	0.353
Riboflavin (mg/day)	1.8 (0.6)	1.7 (0.5)	1.7 (0.5)	0.013
Niacin equivalent	38.4 (9.9)	38.3 (9.6)	36.3 (8.4)	0.012
Vitamin B6 (mg/day)	2.3 (0.9)	2.4 (0.9)	2.3 (0.7)	0.669
Folate (µg/day)	318 (109)	347 (100)	389 (110)	< 0.001
Vitamin B12 (µg/day)	7.5 (3.8)	7.0 (3.0)	6.5 (3.5)	0.001
Vitamin C (mg/day)	130 (79.3)	149 (73.6)	172 (73.5)	< 0.001
Calcium (mg/day)	1057 (446)	1011 (366)	1045 (370)	0.644
Phosphorus (mg/day)	1559 (444)	1566 (396)	1584 (396)	0.477
Magnesium (mg/day)	362.4 (95.2)	392.0 (103.3)	429.4 (105.1)	< 0.001
Potassium (mg/day)	3530 (907)	3654 (966)	3832 (818)	< 0.001
Iron (mg/day)	11.6 (3.4)	12.2 (3.5)	12.8 (3.4)	< 0.001
Zinc (mg/day)	11.5 (3.1)	11.6 (2.9)	11.4 (3.0)	0.528
Selenium (µg/day)	54.3 (16.7)	55.0 (18.4)	52.7 (15.3)	0.315

^1^Values are means and (SD)

^2^P-value for linear trend

^3^Energy percentage of the non-alcoholic energy intake

^4^For individuals with a BMI over 25, weight was estimated as the weight corresponding to a BMI of 25 plus 25% of the remaining weight

^5^Energy-percentage of the total energy intake

In relation to micronutrients, higher adherence to the EAT-Lancet diet was associated with higher intake of beta-carotene, folate, vitamin C, magnesium, potassium, and iron (Table 3). There was an inverse association with retinol equivalents, riboflavin, niacin and vitamin B12. No significant differences were observed for vitamin D, vitamin E, thiamine, vitamin B6, calcium, phosphorus, zinc, or selenium in relation to adherence to the EAT-Lancet diet.

### Nutrient adequacy

Greater adherence to the EAT-Lancet diet was associated with a higher likelihood of meeting the recommended intake (RI) levels for vitamin E, folate, vitamin C, magnesium, potassium, and iron (Table 4). In contrast, vitamin B12 adequacy decreased with increasing adherence to the EAT-Lancet dietary pattern. For the remaining nutrients, no significant associations with dietary adherence were observed. In the total sample, the most common inadequacies in dietary nutrient intake (below RI) were observed for vitamin D (98.5%) and selenium (93.3%).

**Table 4:** Percentage of participants reaching recommended intake (RI) across sex specific tertiles of EAT-Lancet diet score in the H70-study

	Participants reaching recommended intake levels (RI) across tertiles of EAT-Lancet diet score^1^				All
	T1: 10–21	T2: 21–24	T3: 24–38	p-trend^2^	
n	322	284	255		
Nutrients (RI)^3^					
Retinol equivalents (650/750 RE/day)	260 (81%)	230 (81%)	191 (75%)	0.100	681 (79%)
Vitamin D (20 µg/day)	3 (0.93%)	6 (2.11%)	4 (1.57%)	0.492	13 (1.5%)
Vitamin E (9/11 mg/day)	216 (67%)	215 (76%)	219 (86%)	< 0.001	650 (75%)
Thiamine (0.9/1.1 mg/day)	279 (87%)	250 (88%)	224 (88%)	0.650	753 (87%)
Riboflavin (1.6 mg/day)	193 (56%)	164 (58%)	132 (52%)	0.053	489 (57%)
Niacin equivalent (14/18 NE/day)	322 (100%)	284 (100%)	255 (100%)	-	861 (100%)
Vitamin B6 (1.6/1.7 mg/day)	270 (84%)	243 (86%)	227 (89%)	0.08	740 (86%)
Folate (330 µg/day)	120 (37%)	155 (55%)	180 (71%)	< 0.001	455 (53%)
Vitamin B12 (4 µg/day)	295 (92%)	252 (89%)	211 (83%)	0.001	758 (88%)
Vitamin C (95/110 mg/day)	183 (57%)	205 (72%)	219 (86%)	< 0.001	607 (71%)
Calcium (950 mg/day)	169 (53%)	142 (50%)	139 (55%)	0.674	450 (52%)
Phosphorus (520 mg/day)	322 (100%)	284 (100%)	255 (100%)	-	861 (100%)
Magnesium (300/350 mg/day)	209 (65%)	214 (75%)	227 (89%)	< 0.001	650 (75%)
Potassium (3500 mg/day)	145 (45%)	148 (52%)	160 (63%)	< 0.001	453 (53%)
Iron (7/9 mg/day)	287 (89%)	263 (93%)	247 (97%)	0.001	797 (93%)
Zinc (9.3/12.1 mg/day)	185 (58%)	168 (59%)	160 (63%)	0.204	513 (60%)
Selenium (75/85 µg/day)	26 (8.1%)	20 (7.0%)	13 (5.1%)	0.166	59 (6.7%)

^1^Values are n and (%)

^2^P-value for linear trend

^3^Recommended intake (RI) reference values from the Nordic Nutrition recommendations 2023 are reported in parentheses (women/men). For some nutrients, recommendations are the same for women and men

### Biomarkers

Table 5 displays biomarkers that may be influenced by dietary intake, including indicators of glucose and lipid metabolism, as well as homocysteine—an amino acid affected by e.g., B-vitamins such as B12, folate, and B6. A significant negative trend was observed for p-glucose, with increasing adherence to the EAT-Lancet diet. Triglyceride levels were lower, and HDL cholesterol was higher among participants with greater adherence. Similarly, homocysteine concentrations decreased with higher tertiles of adherence, with fewer individuals in the highest adherence group exceeding the reference range. In contrast, no significant associations were observed for total cholesterol, LDL cholesterol, or haemoglobin.

**Table 5:** Biomarkers according to the sex-specific tertiles of the EAT-Lancet diet score in the H70 study

	Biomarkers across tertiles of EAT-Lancet diet score^1^				All
	T1: 10–21	T2: 21–24	T3: 24–38	*p*-trend^2^	
P-Glucose (mmol/L)^3^	6.12 (2)	6.13 (2.1)	5.88 (2.2)	0.024	6.05 (1.2)
S-Cholesterol (mmol/L)^4^	5.45 (1.88)	5.54 (2)	5.6 (2.11)	0.118	5.5 (1.1)
S-Triglycerides (mmol/L) ^4^	1.32 (0.99)	1.19 (1.05)	1.12 (1.11)	< 0.001	1.2 (0.6)
S-HDL-Cholesterol (mmol/L) ^4^	1.69 (0.86)	1.7 (0.92)	1.81 (0.97)	0.008	1.7 (0.5)
S-LDL-Cholesterol (mmol/L) ^4^	3.44 (1.64)	3.54 (1.75)	3.53 (1.84)	0.279	3.5 (1)
B-Haemoglobin (g/L)	144.21 (18.57)	143.85 (19.77)	143.48 (20.85)	0.429	143.9 (11.3)
% Below ref.^5^	14 (4.3%)	14 (4.9%)	11 (4.3%)	0.994	39 (4.5%)
P-Homocysteine (µmol/L)	13.71 (7.52)	12.73 (7.9)	12.16 (8.33)	< 0.001	12.9 (4.6)
% Above ref.^6^	36 (11.2%)	20 (7%)	11 (4.3%)	0.002	67 (7.8%)

^1^Values are means (SD) for continuous variables and n (%) for categorical variables

^2^P-value for linear trend

^3^Adjusted for diabetes prevalence (yes/no)

^4^Adjusted for hypertension (yes/no)

^5^The normal range for haemoglobin was considered > 117 g/L for women and > 134 g/L for men

^6^The normal range for homocysteine was considered < 20 µmol/L

### Sensitivity analysis

After adjusting nutrient intake for total energy intake, adherence to the EAT-Lancet diet remained positively associated with intake of beta-carotene, folate, vitamin C, magnesium, potassium, and iron, as observed in the non-energy adjusted analysis. In addition, thiamine and phosphorus showed positive associations only after adjustment for energy intake (Supplemental Table 2). Inverse associations were observed for retinol equivalents, riboflavin, niacin, and vitamin B12, consistent with the unadjusted model. No significant differences were found for vitamin D, vitamin E, vitamin B6, calcium, zinc, or selenium in relation to diet adherence.

A sensitivity analysis using Average Requirement (AR) values from NNR 2023 was performed alongside the main analysis based on recommended intakes (RI). As expected, a higher proportion of participants met AR compared to RI. The associations between EAT-Lancet diet adherence and nutrient adequacy were consistent in both analyses. Specifically, the proportion of participants meeting AR increased with greater adherence for vitamin E, folate, vitamin C, magnesium, potassium, and iron, while a slight but statistically significant decrease was observed for vitamin B12.

In a sensitivity analysis of biomarker status, we included education as a covariate, which did not alter the associations. Further adjustment for physical activity and smoking showed that the associations for triglycerides and homocysteine remained, while the association for HDL became borderline significant. When additionally adjusting for BMI, only the positive association for homocysteine remained (Supplemental Tables 4–6).

## Discussion

In this cross-sectional study of 70-year-olds in Gothenburg, Sweden, we investigated the relationship between adherence to the EAT-Lancet diet and nutrient adequacy. Higher adherence to the diet was associated with a more favourable micronutrient profile, and with higher intakes of dietary fibre and polyunsaturated fats and lower intakes of saturated fat and alcohol. Although absolute protein intake (g/day) was lower with higher adherence, there was no difference in protein intake when measured per kg/body weight/day. These trends were mirrored in participants’ food choices, with higher adherence linked to greater intake of vegetables, fruits, whole grains, legumes, nuts, and seafood, and lower intake of red and processed meats, eggs, dairy products, added sugar, and potatoes. These dietary shifts align with the EAT-Lancet diet’s emphasis on plant-based foods and suggest improvements in overall diet quality. A higher adherence was also linked to a healthier lifestyle, including higher physical activity, lower BMI, fewer smokers, and higher educational attainment.

In terms of micronutrients, higher adherence to the EAT-Lancet diet was associated with higher intake of beta-carotene, folate, vitamin C, magnesium, potassium, and iron. Conversely, intake of vitamin B12 and retinol equivalents was lower, likely reflecting a lower consumption of animal-derived foods. Other nutrients, including vitamin D, vitamin E, thiamine, and calcium, showed no consistent trends across adherence levels. Across adherence groups, many participants did not meet recommended intake levels for nutrients like vitamin D, and selenium. While this may reflect underlying nutritional challenges or potential underestimation by the dietary assessment method, we believe that other factors may also contribute. In the case of selenium, a key issue is the lack of detailed data on food origin in nutrient databases. Selenium content in food is highly dependent on the concentration of selenium in the soil where the food is grown. In Europe, soils generally contain low levels of selenium, which leads to lower concentrations in locally produced foods [33]. However, this geographical variation is not always accurately represented in nutritional databases, potentially leading to misestimation of intake. In the Swedish national dietary survey “Riksmaten”, the average selenium intake was 32 µg/day among women and 36 µg/day among men [34]—substantially lower than the mean intake of 54 µg/day observed in our study. For vitamin D, dietary intake alone is not a sufficient indicator of deficiency risk, as a significant proportion of vitamin D is synthesized in the skin through exposure to sunlight. The recommended intake assumes that individuals obtain some vitamin D from outdoor activity during the summer [31]. Furthermore, due to the increased risk of vitamin D deficiency among older adults, the Swedish national dietary guidelines recommend a daily supplement of 20 µg of vitamin D for everyone aged 75 years and older [35]. In Sweden, fortification of vitamin D in dairy and plant-based dairy alternatives is mandatory [36]. Voluntary fortification of other nutrients also occurs, but the extent is not well documented.

The EAT-Lancet Commission reported that the EAT-Lancet diet was expected to improve intake of most essential micronutrients, but that vitamin B12 may fall short in diets low in animal-source foods, requiring supplementation or fortification in some cases [1]. A Danish modelling study found that an adapted EAT-Lancet diet met recommended nutrient densities for all micronutrients except vitamin D and iodine in adults aged 6–65 years [37]. However, concerns have been raised about the diet’s ability to provide sufficient micronutrients [17, 20]. Despite the concerns about nutrient adequacy of the EAT-Lancet diet, few cohort studies have examined its nutrient profile in real-world populations. A study by Habumugisha et al., using the Stubbendorff EAT-Lancet score, was performed in a population of predominantly older (55–93 years), poor adults in Kigali, Rwanda, and showed that higher adherence to the EAT-Lancet diet was associated with higher intake of micronutrients [27]. Studies from France concluded that adherence to the EAT-Lancet diet in the age group 18–74 years generally led to an improved intake of several important vitamins and minerals, especially those associated with plant-based foods, using the EAT-Lancet Diet Index (ELD-I) as a scoring system [26, 38]. A study conducted in Germany, used yet another method for quantifying the adherence to the EAT-Lancet diet and found a higher intake of magnesium, vitamin E, folic acid, and vitamin K with higher adherence in 15–18 year olds [25]. A study from Brazil, using the PHDI score in 35–74 year olds found that adherence to the diet was positively associated with most nutrients [39]. While these studies—including our own—indicate that the EAT-Lancet diet can support adequate micronutrient intake across diverse settings, the variation in assessment methods and study populations currently limits the generalizability of these findings.

When accounting for total energy intake, additional positive associations emerged for thiamine and phosphorus—suggesting that some differences may reflect variations in overall food intake rather than dietary quality alone. Persistent inverse associations for vitamin B12, and riboflavin—despite energy adjustment—likely reflect a genuine reduction in foods typically rich in these nutrients, such as meat, dairy, and processed products. These findings highlight the dual benefit and challenge of a plant-forward diet: improvements in several public health-relevant nutrients alongside possible reductions in others reliant on animal sources or fortification.

Although developed with both environmental and human health in mind, the EAT-Lancet diet has been criticized for its potential to fall short in key micronutrients, particularly in vulnerable populations or where intake of animal-source foods is limited foods [17, 26, 40]. In our cohort, even participants in the highest tertile of adherence did not fully meet the reference diet’s targets, with mean adherence scores of 23.1 for women and 21.5 for men (maximum possible score: 42). It should also be noted that few participants met the recommended intake levels for several nutrients in any tertile. Thus, instead of evaluating nutrient adequacy based solely on meeting recommended intake thresholds, it may be more informative to examine the direction and magnitude of change across adherence levels.

Greater adherence to the EAT-Lancet diet was linked not only to lower plasma glucose levels but also to a more favourable lipid profile, characterized by reduced triglyceride concentrations and elevated HDL-cholesterol. This suggests that following the EAT-Lancet dietary pattern may contribute to improved metabolic health, potentially reducing the risk of cardiometabolic diseases.

Importantly, our analysis used a validated EAT-Lancet diet score designed to closely reflect the original reference diet [9]. This scoring approach emphasizes both promoting beneficial food groups and limiting less desirable ones. Because the score reflects diet quality rather than overall intake, it provides a nuanced assessment that is particularly well suited to population-based studies. Energy adjustment also played a key role in revealing diet-quality effects independent of total intake, which may be especially relevant in older adults with lower energy needs.

Finally, this study builds on earlier findings from the H70 cohort, which linked EAT-Lancet diet adherence to markers of better brain health, such as neuroimaging markers of thicker cortex (global and Alzheimer’s disease-signature regions) [15]. Taken together, these results support the growing body of evidence that a sustainable dietary pattern can benefit both planetary and human health, without increasing the risk of micronutrient deficiencies.

### Strengths and limitations

One of the key strengths of this study lies in its thorough dietary assessment, conducted on a systematically selected, well-defined population-based cohort of 70-year-olds. This is the first investigation in Sweden to explore both adherence to the EAT-Lancet dietary recommendations and micronutrient intake among overall healthy older adults. The adherence scoring system used to evaluate compliance with the EAT-Lancet diet has previously shown to strongly reflect the original EAT-Lancet diet when compared with other EAT-Lancet dietary scores [9]. The score has also been associated with lower risk of mortality [9, 10], CVD [12, 41, 42], diabetes [11] and dementia [15, 16]. Nonetheless, the study has some limitations. Given its cross-sectional nature, the dietary data collected through the diet history interview may not accurately capture long-term or habitual intake. While this method generally yields more robust estimates for macronutrients, its reliability tends to be lower for micronutrients such as vitamins and minerals. Still, diet history interviews have advantages, particularly in their ability to capture a wide variety of foods that might be missed with more limited tools like food frequency questionnaires. To help minimize potential measurement errors, trained and experienced research staff conducted the interviews, health assessments, and diagnostic procedures. It is also worth noting that participants born outside of Sweden made up 15.5% of the study sample, a slightly smaller proportion compared to the 19.5% reported among 70-year-olds in Gothenburg [29]. Moreover, individuals who did not speak Swedish (*n* = 53) were excluded from the study), which may limit the generalizability of the findings to populations with demographic profiles similar to those included in the current cohort.

### Future studies

Research is needed that evaluates both reported dietary intake of micronutrients and actual micronutrient status. When assessing the sustainability of diets, it is important to consider not just nutrient intake but also nutrient status, particularly in light of the lower bioavailability of some micronutrients in plant-based foods [43]. Additionally, future studies should examine in more detail how background characteristics, lifestyle factors, and socioeconomic conditions relate to both diet adherence and nutrient status. To better isolate the potential effects of diet itself, it will be important for future research to disentangle the influence of these factors on nutrient status and health outcomes.

## Conclusion

This study provides new insights into the relationship between adherence to the EAT-Lancet diet and nutrient intake among healthy older adults. Higher adherence was associated with a more favourable nutrient profile, including increased intakes of dietary fibre and polyunsaturated fats, and reduced intake of saturated fats. Participants with greater adherence also had higher intake of several vitamins and minerals, without evidence of widespread micronutrient inadequacy. Although intake of vitamin B12 was lower among those with higher adherence, homocysteine concentrations did not indicate functional deficiency, and the prevalence of anaemia was similar across adherence groups. Participants with greater adherence had a more favourable lipid profile.

Overall, these findings suggest that the EAT-Lancet dietary pattern, when applied in real-world settings, can support adequate micronutrient intake in older adults. This reinforces the potential of environmentally sustainable diets to promote health without compromising nutritional adequacy—an important consideration for future dietary guidelines.

## Supplementary Information

Below is the link to the electronic supplementary material.

Supplementary Material 1.

## Data Availability

The dataset used for this study are available from the corresponding author up on reasonable request.
