# Correction: The EAT-Lancet diet in relation to nutrient intake among older adults: insights from the Gothenburg H70 birth cohort study

**DOI:** 10.1186/s12937-025-01211-8

**Published:** 2025-08-29

**Authors:** Anna Stubbendorff, Silke Kern, Lina Rydén, Ingmar Skoog, Jessica Samuelsson

**Affiliations:** 1 Department of Clinical Sciences Malmö, Lund University, Jan Waldenstöms gata 35, Malmö, 21428 Sweden; 2 Neuropsychiatric Epidemiology Unit, Department of Psychiatry and Neurochemistry, Institute of Neuroscience and Physiology, Sahlgrenska Academy, Centre for Ageing and Health (AGECAP) at the University of Gothenburg, Wallinsgatan 6, Mölndal, 43139 Sweden; 3 Region Västra Götaland, Department of Neuropsychiatry, Sahlgrenska University Hospital, Gothenburg, Sweden

**Correction: Nutr J 24, 124 (2025)**

**https://doi.org/10.1186/s12937-025-01193-7**

Following publication of the original article [1], the author reported that the article title should be updated from “The EAT-Lancet diet in relation nutrient intake among older adults: Insights from the Gothenburg H70 Birth Cohort Study” to “The EAT-Lancet diet in relation **to** nutrient intake among older adults: Insights from the Gothenburg H70 Birth Cohort Study”

The original article has been updated.
