# Supplementary material for: The EAT-Lancet diet in relation to nutrient intake among older adults: insights from the Gothenburg H70 birth cohort study
Source: Nutr J. 2025 Aug 8;24:124. doi: 10.1186/s12937-025-01193-7 (PMC12335021; doi:10.1186/s12937-025-01193-7)
Supplement: Supplementary file 1 — Supplementary Material 1. [file 12937_2025_1193_MOESM1_ESM.docx]

**Online supplementary material**

Supplement to: Stubbendorff A, et al. The EAT-Lancet diet in relation nutrient intake among older adults: Insights from the Gothenburg H70 Birth Cohort Study.

**Supplemental figure 1.** Flowchart of included participants





**Supplemental Table 1.** Formulation of the EAT-Lancet diet Index from Stubbendorff [1].

| **Component** | **Recommended intake (interval)** | **Score of 3** | **Score of 2** | **Score of 1** | **Score of 0** |
| --- | --- | --- | --- | --- | --- |
| Vegetables | 300 (200-600) g/d | >300 g/d | 200-300 g/d | 100-200 g/d | <100 g/d |
| Fruits | 200 (100-300) g/d | >200 g/d | 100-200 g/d | 50-100 g/d | <50 g/d |
| Unsaturated oils | 40 (20-80) g/d | >40 g/d | 20-40 g/d | 10-20 g/d | <10 g/d |
| Legumes | 75 (0-150) g/d | >75 g/d | 37.5-75 g/d | 37.5-18.75 g/d | <18.75 g/d |
| Nuts | 50 (0-100) g/d | >50 g/d | 25-50 g/d | 12.5-25 g/d | <12.5 g/d |
| Whole grains | 232 g/d | >232 g/d | 116-232 g/d | 58-116 g/d | <58 g/d |
| Fish | 28 (0-100) g/d | >28 g/d | 14-28 g/d | 7-14 g/d | <7 g/d |
| Beef and lamb | 7 (0-14) g/d | <7 g/d | 7-14 g/d | 14-28 g/d | >28 g/d |
| Pork | 7 (0-14) g/d | <7 g/d | 7-14 g/d | 14-28 g/d | >28 g/d |
| Poultry | 29 (0-58) g/d | <29 g/d | 29-58 g/d | 58-116 g/d | >116 g/d |
| Eggs | 13 (0-25) g/d | <13 g/d | 13-25 g/d | 25-50 g/d | >50 g/d |
| Dairy | 250 (0-500) g/d | <250 g/d | 250-500 g/d | 500-1000 g/d | >1000 g/d |
| Tubers | 50 (0-100) g/d | <50 g/d | 50-100 g/d | 100-200 g/d | >200 g/d |
| Added sugar | 31 (0-31) g/d | <31 g/d | 31-62 g/d | 62-124 g/d | >124 g/d |
| ^1^Each component contributed 0-3 points resulting in a score ranging from 0 to 42 points | | | | | |

1. Stubbendorff, A., et al., Development of an EAT-Lancet index and its relation to mortality in a Swedish population. The American Journal of Clinical Nutrition, 2021.

**Supplemental table 2.** Nutrient intake per day adjusted for total energy intake (kcal/day) across sex specific tertiles of EAT-Lancet diet score H70-study.

|  | **Nutrient intake across tertiles of adherence**  **to the EAT-Lancet score^1^** | | | |
| --- | --- | --- | --- | --- |
|  | **T1: 10-21** | **T2: 21-24** | **T3: 24-38** | **p-trend^2^** |
| Retinol Equivalent | 1192 (697) | 1125 (618) | 1072 (650) | 0.018 |
| Beta-Carotene (µg/day) | 2754 (1964) | 3442 (2473) | 4446 (4020) | <0.001 |
| Vitamin D (µg/day) | 8.5 (3.8) | 9.1 (4.3) | 9.0 (4.2) | 0.122 |
| Vitamin E (mg/day) | 15.2 (15.5) | 16.4 (15.0) | 15.8 (11.1) | 0.610 |
| Thiamine (mg/day) | 1.3 (0.4) | 1.4 (0.4) | 1.4 (0.4) | 0.012 |
| Riboflavin (mg/day) | 1.8 (0.6) | 1.7 (0.5) | 1.7 (0.5) | 0.023 |
| Niacin Equivalent | 38.1 (9.9) | 38.3 (9.6) | 36.7 (8.4) | 0.019 |
| Vitamin B6 (mg/day) | 2.3 (0.9) | 2.4 (0.9) | 2.3 (0.7) | 0.286 |
| Folate (µg/day) | 316 (109) | 347 (100) | 392 (110) | <0.001 |
| Vitamin B12 (µg/day) | 7.4 (3.8) | 7.0 (3.0) | 6.6 (3.5) | 0.003 |
| Vitamin C (mg/day) | 129 (79) | 149 (74) | 173 (73) | <0.001 |
| Calcium (mg/day) | 1046 (446) | 1011 (366) | 1060 (370) | 0.679 |
| Phosphorus (mg/day) | 1544 (444) | 1566 (396) | 1603 (396) | 0.005 |
| Magnesium (mg/day) | 359 (95) | 392 (103) | 434 (105) | <0.001 |
| Potassium (mg/day) | 3500 (907) | 3654 (966) | 3870 (818) | <0.001 |
| Iron (mg/day) | 11.5 (3.4) | 12.2 (3.5) | 13.0 (3.4) | <0.001 |
| Zinc (mg/day) | 11.4 (3.1) | 11.6 (2.9) | 11.5 (3.0) | 0.616 |
| Selenium (µg/day) | 53.9 (16.7) | 55.0 (18.4) | 53.2 (15.3) | 0.626 |
|  |  |  |  |  |
| 1. Values are means and (SD).  2. P-value for linear trend. | | | | |

**Supplemental table 3.** Percentage of participants reaching average requirement (AR) across sex specific tertiles of EAT-Lancet diet score in the H70-study.

|  | **Participants reaching recommended intake levels (RI) across tertiles of EAT-Lancet diet score^1^** | | | |  |  |
| --- | --- | --- | --- | --- | --- | --- |
|  | **T1: 10-21** | **T2: 21-24** | **T3: 24-38** | **p-trend**^2^ |  | **all** |
|  |  |  |  |  |  |  |
| n | 322 | 284 | 255 |  |  |  |
| Nutrients (AR) |  |  |  |  |  |  |
| Retinol Equivalents (510/590 RE/day) | 293 (91.0%) | 260 (91.5%) | 232 (91.0%) | 0.99 |  | 785 (91.2%) |
| Vitamin D (7.5 µg/day) | 188 (58.4%) | 168 (59.2%) | 151 (59.2%) | 0.835 |  | 507 (58.9%) |
| Vitamin E (8/9 mg/day) | 267 (82.9%) | 250 (88.0%) | 240 (94.1%) | <0.001 |  | 757 (87.9%) |
| Thiamine (0.65/0.75 mg/day) | 318 (98.8%) | 281 (98.9%) | 253 (99.2%) | 0.595 |  | 852 (99.0%) |
| Riboflavin (1.3 mg/day) | 273 (84.8%) | 240 (84.5%) | 209 (82.0%) | 0.375 |  | 722 (83.9%) |
| Niacin Equivalent (12/15 NE/day) | 322 (100%) | 284 (100%) | 255 (100%) | . |  | 861 (100%) |
| Vitamin B6 (1.3/1.5 mg/day) | 304 (94.4%) | 271 (95.4%) | 248 (97.3%) | 0.105 |  | 823 (95.6%) |
| Folate (250 µg/day) | 242 (75.2%) | 238 (83.8%) | 237 (92.9%) | <0.001 |  | 717 (83.3%) |
| Vitamin B12 (3.2 µg/day) | 312 (96.9%) | 274 (96.5%) | 237 (92.9%) | 0.028 |  | 823 (95.6%) |
| Vitamin C (75/90 mg/day) | 224 (69.6%) | 243 (85.6%) | 233 (91.4%) | <0.001 |  | 700 (81.3%) |
| Calcium (750 mg/day) | 243 (75.5%) | 219 (77.1%) | 200 (78.4%) | 0.398 |  | 662 (76.9%) |
| Phosphorus (420 mg/day) | 322 (100%) | 284 (100%) | 255 (100%) | . |  | 861 (100%) |
| Magnesium (240/280 mg/day) | 291 (90.4%) | 263 (92.6%) | 249 (97.6%) | 0.001 |  | 803 (93.3%) |
| Potassium (2800 mg/day) | 256 (79.5%) | 228 (80.3%) | 236 (92.5%) | <0.001 |  | 720 (83.6%) |
| Iron (6/7 mg/day) | 310 (96.3%) | 277 (97.5%) | 253 (99.2%) | 0.029 |  | 840 (97.6%) |
| Zinc (7.7/10.1 mg/day) | 270 (83.9%) | 234 (82.4%) | 213 (83.5%) | 0.893 |  | 717 (83.3%) |
| Selenium (60/70 µg/day) | 71 (22.0%) | 65 (22.9%) | 56 (22.0%) | 0.995 |  | 192 (22.3%) |
|  |  |  |  |  |  |  |
| 1. Values are n and (%).  2. P-value for linear trend3. Average requirement (AR) reference values from the Nordic Nutrition recommendations 2023 are reported in parentheses (women/men). For some nutrients, recommendations are the same for women and men. | | | | | | |

**Supplemental table 4.** Biomarkers according to the sex-specific tertiles of the EAT-Lancet diet score in the H70 study, adjusted for education.

|  | **Biomarkers across tertiles of**  **EAT-Lancet diet score^1^** | | | |
| --- | --- | --- | --- | --- |
|  | **T1: 10-21** | **T2: 21-24** | **T3: 24-38** | **p-trend^2^** |
| P-Glucose (mmol/L)^3^ | 6.11 (2) | 6.12 (2.1) | 5.9 (2.2) | 0.048 |
| S-Cholesterol (mmol/L)^4^ | 5.45 (1.9) | 5.54 (2) | 5.61 (2.1) | 0.088 |
| S-Triglycerides (mmol/L) ^4^ | 1.31 (1) | 1.19 (1) | 1.14 (1.1) | <0.001 |
| S-HDL-Cholesterol (mmol/L) ^4^ | 1.69 (0.9) | 1.7 (0.9) | 1.81 (1) | 0.010 |
| S-LDL-Cholesterol (mmol/L) ^4^ | 3.44 (1.6) | 3.54 (1.7) | 3.54 (1.8) | 0.218 |
| B-Haemoglobin (g/L) | 144.21 (18.57) | 143.85 (19.77) | 143.48 (20.85) | 0.449 |
| % below ref.^5^ | 4.5% | 5.0% | 4.1% | 0.825 |
| P-Homocysteine (µmol/L) | 13.71 (7.52) | 12.73 (7.9) | 12.16 (8.33) | <0.001 |
| % above ref.^6^ | 10.7% | 7% | 4.4% | 0.005 |
| 1. Values are means (SD) for continuous variables and n (%) for categorical variables.  2. P-value for linear trend  3. Adjusted for diabetes prevalence (yes/no)  4. Adjusted for hypertension (yes/no)  5. The normal range for haemoglobin was considered >117 g/L for women and >134 g/L for men  6. The normal range for homocysteine was considered < 20 µmol/L. | | | | |

**Supplemental table 5.** Biomarkers according to the sex-specific tertiles of the EAT-Lancet diet score in the H70 study, adjusted for education, physical activity and smoking status.

|  | **Biomarkers across tertiles of**  **EAT-Lancet diet score^1^** | | | |
| --- | --- | --- | --- | --- |
|  | **T1: 10-21** | **T2: 21-24** | **T3: 24-38** | **p-trend^2^** |
| P-Glucose (mmol/L)^3^ | 6.03 (2.0) | 6.2 (2.1) | 5.92 (2.2) | 0.315 |
| S-Cholesterol (mmol/L)^4^ | 5.49 (1.9) | 5.51 (2.0) | 5.6 (2.1) | 0.254 |
| S-Triglycerides (mmol/L) ^4^ | 1.27 (1.0) | 1.2 (1.0) | 1.15 (1.1) | 0.012 |
| S-HDL-Cholesterol (mmol/L) ^4^ | 1.72 (0.9) | 1.69 (0.9) | 1.8 (1.0) | 0.065 |
| S-LDL-Cholesterol (mmol/L) ^4^ | 3.47 (1.7) | 3.51 (1.8) | 3.53 (1.9) | 0.525 |
| B-Haemoglobin (g/L) | 144.41 (19.0) | 143.76 (20.1) | 143.16 (20.9) | 0.202 |
| P-Homocysteine (µmol/L) | 13.49 (7.5) | 14 (4.9) | 11 (4.3) | <0.001 |
| 1. Values are means (SD) for continuous variables and n (%) for categorical variables.  2. P-value for linear trend  3. Adjusted for diabetes prevalence (yes/no)  4. Adjusted for hypertension (yes/no)  5. The normal range for haemoglobin was considered >117 g/L for women and >134 g/L for men  6. The normal range for homocysteine was considered < 20 µmol/L. | | | | |

**Supplemental table 6.** Biomarkers according to the sex-specific tertiles of the EAT-Lancet diet score in the H70 study, adjusted for education, physical activity, smoking status and BMI.

|  | **Biomarkers across tertiles of**  **EAT-Lancet diet score^1^** | | | |
| --- | --- | --- | --- | --- |
|  | **T1: 10-21** | **T2: 21-24** | **T3: 24-38** | **p-trend^2^** |
| P-Glucose (mmol/L)^3^ | 6.0 (2.0) | 6.19 (2.1) | 5.95 (2.19) | 0.649 |
| S-Cholesterol (mmol/L)^4^ | 5.51 (1.9) | 5.54 (2.0) | 5.58 (2.1) | 0.498 |
| S-Triglycerides (mmol/L) ^4^ | 1.25 (0.9) | 1.19 (1.0) | 1.17 (1.0) | 0.102 |
| S-HDL-Cholesterol (mmol/L) ^4^ | 1.74 (0.8) | 1.7 (0.9) | 1.77 (0.9) | 0.419 |
| S-LDL-Cholesterol (mmol/L) ^4^ | 3.49 (1.7) | 3.53 (1.8) | 3.53 (1.9) | 0.629 |
| B-Haemoglobin (g/L) | 144.38 (19.0) | 143.6 (20.0) | 143.39 (20.9) | 0.304 |
| P-Homocysteine (µmol/L) | 13.47 (7.6) | 12.93 (7.9) | 12.15 (8.3) | 0.001 |
| 1. Values are means (SD) for continuous variables and n (%) for categorical variables.  2. P-value for linear trend  3. Adjusted for diabetes prevalence (yes/no)  4. Adjusted for hypertension (yes/no)  5. The normal range for haemoglobin was considered >117 g/L for women and >134 g/L for men  6. The normal range for homocysteine was considered < 20 µmol/L. | | | | |

**References:**

1. Stubbendorff, A., et al., *Development of an EAT-Lancet index and its relation to mortality in a Swedish population.* The American Journal of Clinical Nutrition, 2021.
